# Examining Whether Semantic Cues Can Affect Felt Heaviness When Lifting Novel Objects

**DOI:** 10.5334/joc.93

**Published:** 2020-01-31

**Authors:** Caitlin Elisabeth Naylor, T. J. Power, Gavin Buckingham

**Affiliations:** 1 Sport and Health Sciences, College of Life and Environmental Sciences, University of Exeter, Exeter, UK

**Keywords:** Action and perception, Multi-sensory perception, Semantics

## Abstract

It is well established that manipulations of low-level stimulus properties unrelated to mass can impact perception of heaviness, the most famous example being the size-weight illusion whereby small objects feel heavier than equally-weighted larger objects. Interestingly, manipulations of high-level cues such as material have also induced weight illusions, highlighting that cognitive expectations alone are enough to create illusory weight differences. Less is known, however, about what type of cognitive expectations can influence perception of heaviness. As labels are often used to signify the heaviness of objects, this study examined whether semantic cues could induce a novel weight illusion. Participants lifted equally-sized and equally-weighted sets of objects labelled as ‘light’ and ‘heavy’ and reported their perceived heaviness both prior to and after lifting. Fingertip forces were also measured to understand how semantic cues may influence sensorimotor prediction. The labels clearly affected pre-lift-off expectations of heaviness. By contrast, we found no effect of these labels on the perceived heaviness of objects, nor on the forces used to grip and lift them on early trials. In other words, we find no evidence that semantic cues affect perception or action enough to induce a novel weight illusion. These findings suggest that the explicit expectations created by the labels did not dominate the implicit expectations created by the equal sizes of the objects, highlighting the segregated nature of cognitive expectations and their variable influences on perception and action.

## Introduction

Our experience of how heavy an object feels is notoriously fallible, as this percept is affected by a range of factors unrelated to its mass. For example, in the size-weight illusion small objects feel up to 50% heavier than larger objects with the same mass (Charpentier, 1891; Nicolas et al., 2012). The mechanism underpinning this effect, and its utility, are still far from certain (Ernst, 2009; Zhu and Bingham, 2011; Buckingham, 2014; Dijker, 2014), but one popular explanation suggests that the illusory weight differences which characterize the SWI reflect the way in which expectations (priors) are integrated with sensory input in this particular context. In short, because individuals have reasonably strong expectations that small objects will, on average, be less-heavy than larger versions of the same object, they subsequently experience a contrast with that prior belief. Evidence for the role of prior experience in subsequent perception of object weight comes from studies by Flanagan and colleagues (Flanagan et al., 2008), who allowed participants extensive experience with inverted-density objects, reducing and eventually reversing the magnitude of the SWI experienced with identically-weighted stimuli (see also Nakatani, 1985). Other, more recent, variants of this explanation suggest that expected density is the variable integrated with sensory inputs related to object mass to yield the experience of an object’s heaviness (Peters et al., 2016). Consistent across both of these explanations, however, is that cognitive factors are modulating the input of the sensory organs to modulate hedonic experience of an object’s heaviness.

As alluded to above, a range of factors can affect how heavy an object feels, with studies isolating the top-down effects on the SWI suggesting that approximately half of its magnitude can be ascribed to a lifters’ expectations (Buckingham, 2014; Buckingham et al., 2011a; Buckingham and Goodale, 2010a). The other half of the full strength SWI experienced ‘naturally’ can be at least partially accounted for by factors such as moment of inertia and hand aperture (Amazeen and Turvey, 1996; Flanagan and Bandomir, 2000). Curiously, other similar effects have been reported where factors other than object volume are manipulated to induce a weight illusion. The most famous of these – the material-weight illusion, describes how objects which appear to be made from low-density material (e.g., polystyrene) feel heavier than objects of the same mass which appear to be made from a high-density material (e.g., metal). First described by Seashore (1899), this effect has been examined in a range of contexts, with studies indicating that it appears to operate through an independent mechanism from the SWI (Buckingham et al., 2009; Vicovaro and Burigana, 2017). As the association between material cues and object mass is arbitrary (i.e., unable to be deduced from low-level cues such as retinal image size), the material-weight illusion is a clear example of an illusion induced solely by cognitive expectations.

Of course, an individual’s expectations about object properties can be influenced by a range of implicit and explicit factors. For example, if a box was labelled as ‘heavy’, this would likely influence a lifter’s expectations at some level – namely the way in which they might lift it and the way in which they might articulate its likely heaviness to another. However, it is well-understood that one’s conscious knowledge is easily dissociated from the predictive application of fingertip forces (Flanagan and Beltzner, 2000; Grandy and Westwood, 2006) and gross motor output (Buckingham et al., 2014). Furthermore, recent work has suggested that the expectations which induce weight illusions are also distinct from consciously accessible expectations. Buckingham and MacDonald (2016) showed that a small sports ball which was expected to be heavy prior to lifting still feeling heavier than a large sports ball of the same mass which was expected to be relatively light prior to lifting (cf. typical weight illusion scenarios, where the small objects are expected to be less-heavy than the large objects at the outset of the study). It is thus still far from clear the level at which a cue to heaviness must be in order to make an object feel lighter or heavier than it actually is. To date, weight illusions have certainly not been confined to ‘low-level’ stimulus manipulations – misperceptions of heaviness have been induced with matched-weight stimuli which vary in object identity (cricket vs tennis balls; Buckingham & MacDonald, 2016), importance (books and USB memory drives; Schneider et al., 2011, 2015) and even gender (male vs female dolls; Dijker, 2008).

The main aim of this study was to evaluate whether semantic cues (i.e., labels) can affect perceptions of heaviness to a sufficient degree to induce a novel weight illusion, akin to those outlined above. In a short pilot study undertaken as an undergraduate thesis project, 18 participants lifted light and heavy exemplars of otherwise-identical-looking objects of the same mass which were labelled as ‘heavy’ and ‘light’ multiple times. After each lift, participants rated the felt heaviness of the object they just interacted with on an arbitrary numerical scale, and these ratings were normalized to a z-distribution. This pilot study found compelling-enough evidence that participants experienced objects labelled as ‘light’ to feel heavier than objects labelled as ‘heavy’ (i.e., a main effect of the label; *F*(1,17) = 6.97, *p* = .017) with an appropriately-sized effect (Cohen’s *d* = 0.62). It is worth noting that the effect size for labelling illusion is somewhat (and appropriately) smaller than that demonstrated in a previous study (Buckingham and MacDonald, 2016) for an equivalent test across a tennis ball (normally 55-g) and cricket ball (normally 158-g) adjusted to have the same weight as one another (Cohen’s *d* = 0.86). The outcomes from this pilot study, in combination with the growing body of factors found to induce weight illusions, suggests that this would be an appropriate target for a formal pre-registered experiment.

A secondary goal of this study was to evaluate the degree to which fingertip forces are affected by semantic cues. Because humans apply fingertip force in a predictive way, measurements of grip and load force rates in lifts of novel object typically reflect the lifters expectations of the object’s weight. Thus, in SWI paradigms the large object is lifted at a higher rate of force than the small object, and in MWI paradigms the dense-looking object is lifted at a higher rate of force than the non-dense looking object (Gordon et al., 1991; Buckingham et al., 2009). Through largely-implicit processes, these initial sensorimotor predictions are rapidly refined over the course of a few interactions, and fingertip forces start reflecting the actual, rather than expected mass of the objects (Flanagan and Beltzner, 2000; Grandy and Westwood, 2006). Although no study has examined whether semantic cues drive sensorimotor prediction, it has been shown that the content of words spoken to participants while they are holding object can modulate their fingertip forces, such that individuals will tend to squeeze the handle of a force transducer slightly tighter upon hearing a verb than they would upon hearing a noun (Frak et al., 2010; Silva et al., 2018). Given that labels on objects largely exist to warn potential lifters about the weight of the objects (e.g., the ‘heavy luggage’ label regularly attached to suitcases prior to air travel), it seems likely that an object labelled ‘heavy’ will be lifted with more force than one labelled ‘light’, and that this propensity to use these semantic cues to guide fingertip forces will attenuate with repeated lifts.

### Hypotheses

1. On average, objects labelled as ‘heavy’ would feel less-heavy than objects labelled as ‘light’, and this illusion would be present across all trials.
2. Initially (i.e., during the first lifts), objects labelled as ‘heavy’ would be picked up with a higher rate of force than objects labelled as ‘light’.

## Method

### Sample size

An a-priori power analysis calculated in G*Power (v3.1.9.2) suggested that a sample size of 36 would yield a power of .95 to detect an effect equivalent to that found in the pilot study outlined above (a Cohen’s d of 0.62 with a 2-tailed paired-sample t-test). This effect size, while classically defined as a medium effect, is smaller than has been shown in other similar weight illusion studies (Buckingham et al., 2009; Buckingham and MacDonald, 2016), and thus represents a suitably conservative value. In total, 38 participants (16 males, 22 females; mean age = 26.7 years, range = 18–56 years, SD = 11.6) were recruited for the experiment. All participants had normal or corrected-to-normal vision and 33 reported being right-handed. Participants provided written informed consent to participate in the study, and all procedures were approved by the local research ethics board.

### Materials

**Figure 1:**
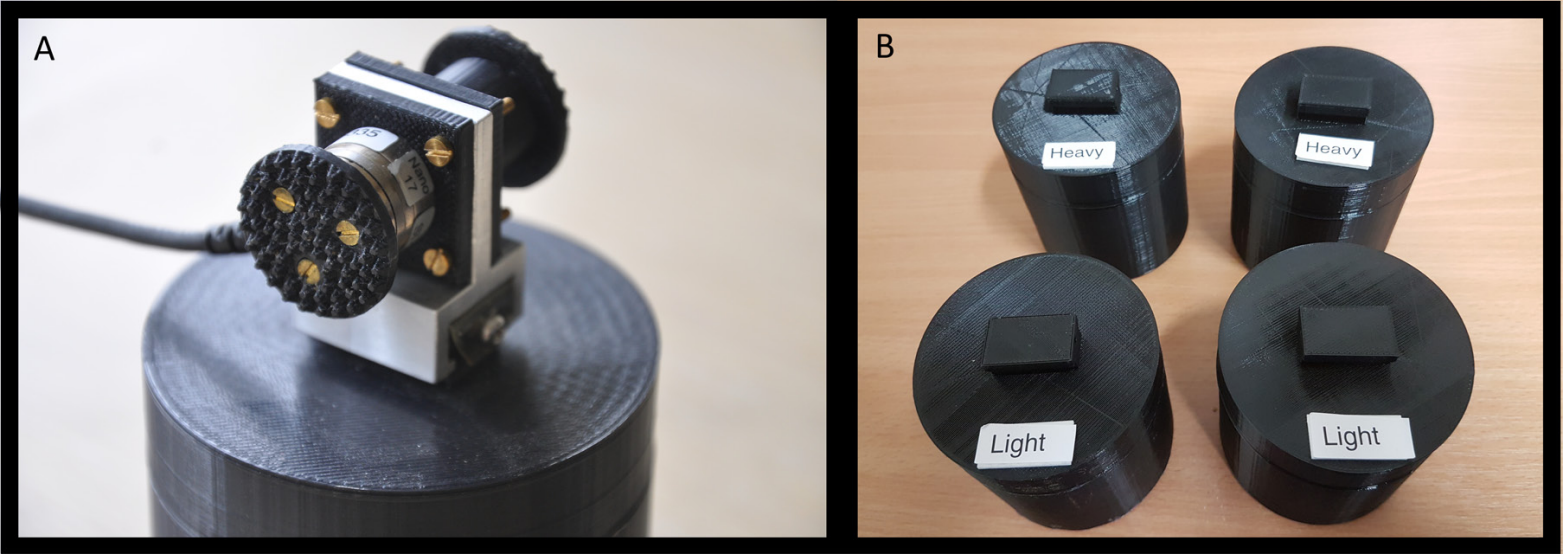
**A.** The force transducer handle which participants use to lift the objects, attached to the unlabelled practice cylinder. **B.** The labelled test cylinders lifted by participants during the experimental trials.

Participants lifted and judged the weight of the 7.5 cm tall, 7.5 cm diameter black PLA cylinders used in the pilot work. The cylinders, which were created on an Ultimaker^2^ 3d printer, have rubberised feet to dampen auditory cues to heaviness, and were filled with packing foam and lead shot such that the heavier pair of cylinders weighed 486 g and the lighter pair of cylinders weighed 356 g. These cylinders had a t-shaped mount in the centre of their top surface which allowed for the quick attachment and removal of a custom-build aluminium and plastic handle containing a single ATI Nano17 6-axis force transducer (which, itself, weighed 36 g). The transducer, and its opposing surface, were covered in a textured pad 25 mm in diameter. These grasp pads were 50 mm apart from one another, facilitating a comfortable precision grip with the thumb and index finger (Figure 1A). On the top surface of these cylinders, oriented toward the participant, labels with the text ‘Light’ and ‘Heavy’ printed on white paper in size 20 Arial font were affixed with double-sided tape (Figure 1B). Both the 486 g and 356 g sets’ objects had these labels, for a total of four experimental objects.

### Procedures

This experiment followed a well-established protocol for weight illusion studies (Buckingham et al., 2009, 2016a, 2016b), with perceptual ratings and fingertip force measurements taken on every trial. On each trial, participants sat in front of a table in a height-adjustable chair with their eyes closed and their dominant hand flat on the table surface. An object was placed in front of them, and an auditory cue signalled the participant to open their eyes and reach out to pick up the object on the grasp pads with the thumb and index finger of their preferred hand. They were instructed to lift the object in a smooth, controlled, and confident manner a short distance off the table surface, and to hold the object steady until a second auditory cue (four seconds after the first) signalled them to put it down. Participants then gave an unconstrained numerical rating for how heavy the object felt on that particular trial (i.e., an absolute magnitude estimation; Zwislocki & Goodman, 1980). It was emphasised to participants through a semi-standardized briefing that there were no upper or lower limits to their scale, and they should simply report their experience during the previous lift. Participants lifted each of the four objects 10 times apiece in one of four pseudo-randomized orders generated with the Microsoft Excel rand() function, two of which were the inverse of the other two with respect to the label attached to the object. In order to allow for the evaluation of sensorimotor prediction during the initial lifts in a balanced way, independent from the effect of physical mass differences, we standardized the initial order in which the objects were presented. In two of these orders, the first two lifts were of the object labelled as ‘heavy’ from the heavy (486 g) set, followed by the object labelled as ‘light’ from the heavy set, and in two of these orders the first two lifts were the object labelled as ‘light’ from the heavy set, followed by the object labelled as ‘heavy’ from the heavy set.

Before starting the experiment, participants were given five practice trials with an unlabelled 486 g practice cylinder of the same dimensions as the test cylinders, to ensure their lifting technique was appropriate, calibrate their perceptual scales, and ensure their fingertip forces during the critical first lifts were not unduly affected by interactions prior to entering the laboratory (i.e., a washout phase). After the practice trials, participants were then presented with the four objects one at a time, with eyes closed in between each presentation to avoid kinematic cues to heaviness, in a randomized sequence and asked to rate how heavy they thought it would feel based solely on their visual appearance (i.e., without touching them) using their numerical scale. Following these pre-lift-off ratings of expected heaviness, they started the experimental trials proper.

### Data reduction and analyses

Prior to data reduction, two participants were excluded due to repeatedly lifting the objects incorrectly and failing to follow the standard procedure, leaving a total of 36 datasets for analysis. The perceptual ratings were transformed into z scores within-subject, in order to account for individual differences in the rating scale used, based on the mean and standard deviation of their ratings given during the experimental trials. As the magnitude of weight illusions tends to remain constant across repeated lifts (Buckingham et al., 2009; Buckingham and Goodale, 2010b), these z scores were averaged across all 10 trials then examined in a 2 (label) × 2 (mass) repeated measures ANOVA.

The forces recorded by the transducer were pre-processed with custom Matlab scripts (found here: https://sites.google.com/site/obintlab/wiki/data-processing). The force vector orthogonal to the grasp pads was defined as grip force, and the forces parallel to the surface of the grasp pad were vector-summed and defined as load force. These force traces were filtered with a 14Hz Butterworth Filter and differentiated with a 5-point central difference equation to yield their rates of change. The peak values (all of which were visually confirmed on a trial by trial basis) of grip and load force rates (pGFR and pLFR respectively) were taken as the primary sensorimotor variables of interest. As fingertip forces in the context of weight illusion paradigms adapt on a trial by trial basis (Grandy and Westwood, 2006; Buckingham et al., 2009), the pGFR and pLFR applied to the cylinders on the first two lifts of the experimental trials were examined, which only varied across the factor of ‘label’ in all trial orders, and were thus examined with a simple paired-sample t-test (Hypothesis 2). Due to focussing on the first two lifts, one participant was excluded from force analysis due to experimenter error (the incorrect object was lifted on trial 2), leaving a total sample of 35 for force analysis. In addition to the statistical analysis, the force rates applied to all the objects on each trials were visualized.

To examine whether the labels influenced explicit, cognitive, prior expectations of heaviness, the pre-lift-off ratings were also examined in a 2 (label) × 2 (mass) repeated measures ANOVA. Here, a main effect of label would suggest that semantic cues affect how heavy participants expect each object to be. As the objects had no visible cues to mass beyond their label, no main effect of mass, nor an interaction between mass and weight, would be expected.

Finally, associations between these individual values and those derived from the perceptual heaviness ratings and fingertip force rate measures were examined with a series of Pearson’s correlations. Finding a significant correlation between the pre-lift-off heaviness ratings and the subsequent measurements would strongly suggest that these explicit prior expectations play a causal role in any perceptual and sensorimotor effects observed.

Statistical analyses were performed in JAMOVI v1.1.6.0. Data can be found at https://osf.io/69dxj/.

### Deviations from preregistered protocol in the Stage 1 report

1. Object weight was incorrectly reported in the stage 1 protocol as 450 g and 380 g, as opposed to the actual stimuli weights of 486 g and 356 g.
2. The first two lifts were taken from the heavy set of stimuli, as opposed to the light set (as reported in the stage 1 report). Accordingly, the practice object weighed the same as the heavy set of stimuli (486 g), rather than the 380 g reported in the stage 1 report.
3. The fingertip force data were filtered at 14Hz, rather than the reported 50Hz, for consistency with the senior author’s prior work in the field.
4. Statistical analyses were performed in JAMOVI v1.1.6.0.

## Results

### Preregistered analyses

**Figure 2:**
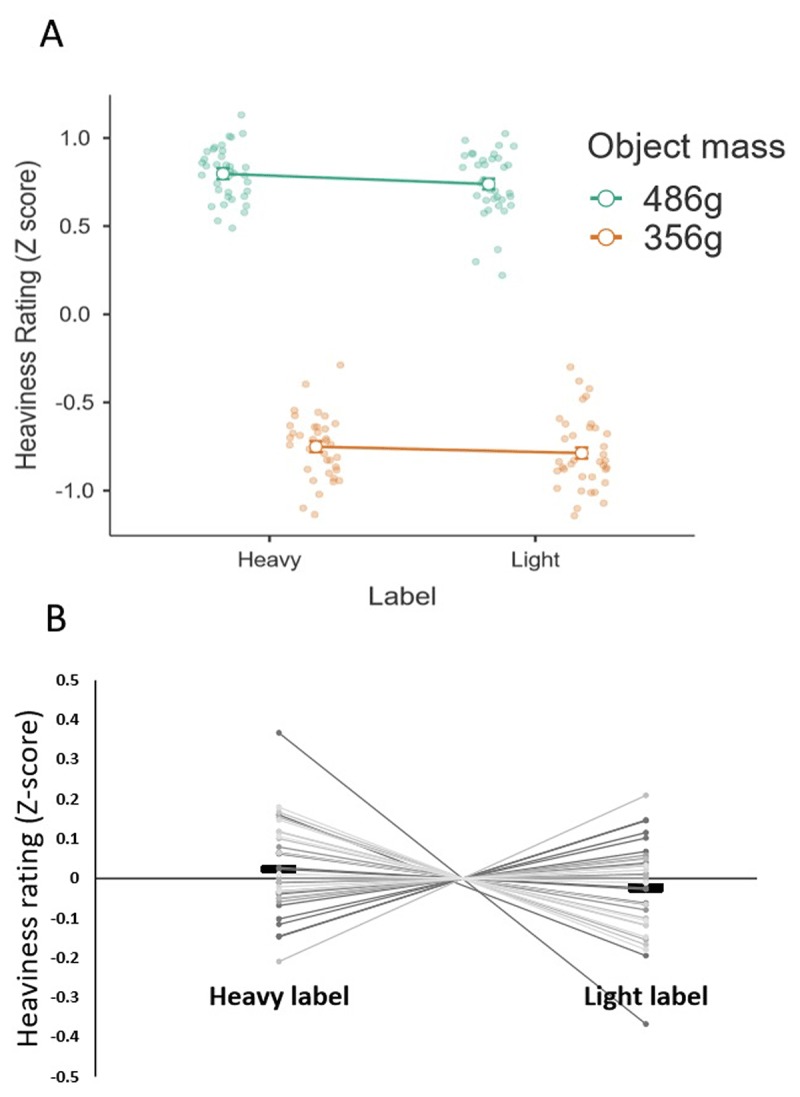
**A.** The average perceptual ratings of heaviness, normalized to a z distribution, for each of the four lifted objects. **B.** The main effect of object label (averaged across mass), visualized for individual participants’ average perceptual ratings of heaviness normalised to a z distribution. The solid rectangle represent the group average.

In terms of the reported perceptual experience of heaviness, the expected main effect of mass was found, confirming that participants experienced and reported a difference in physical mass between the stimuli (*F*(1, 35) = 2161.27, *p* < .001, \eta _p^2 = .984; Figure 2A). By contrast, there was no significant main effect of label (*F*(1, 35) = 1.56, *p* = .219, \eta _p^2 = .043). Therefore, in contrast to the prediction of our first hypothesis, there was no difference in the perceived heaviness between the objects labelled as ‘light’ and objects labelled as ‘heavy’; in other words the objects’ labels did not induce a novel weight illusion (illustrated in Figures 2A and 2B). No label x mass interaction was found (*F*(1, 35) = .11, *p* = .743, \eta _p^2 = .003).

**Figure 3:**
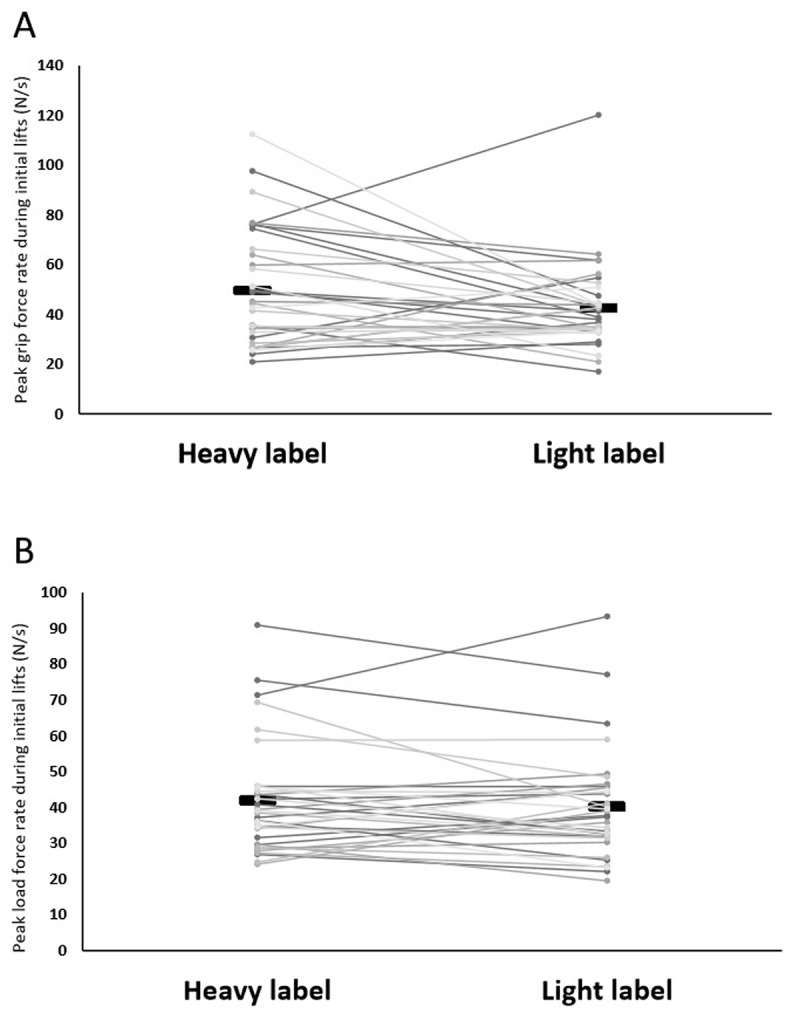
**A.** The peak grip force rate applied on the initial lifts of the objects labelled as ‘heavy’ and ‘light’ and **B.** the load force rate applied on the initial lifts of the objects labelled as ‘heavy’ and ‘light’, for each individual. The solid rectangles represent the group averages.

**Figure 4:**
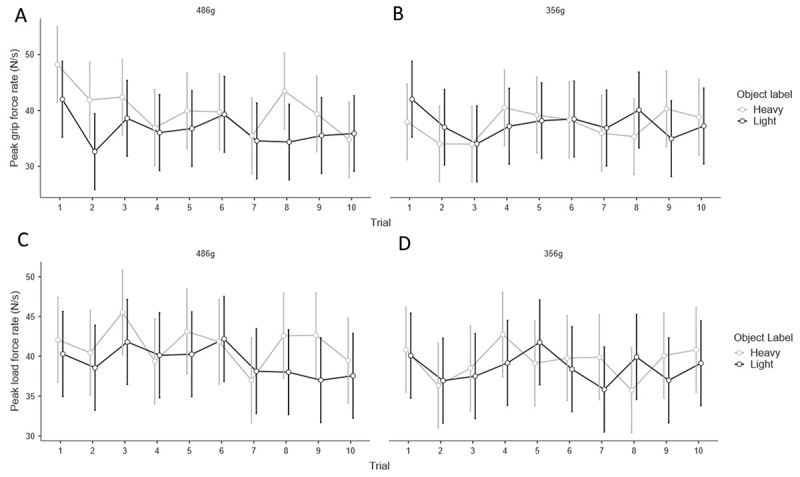
**A.** Peak grip force rate across all lifts of the 486 g objects and **B.** the 356 g objects labelled as ‘heavy’ and ‘light’. **C.** The peak load force rates across all lifts of the 486g objects and **D.** the 356 g objects labelled as ‘heavy’ and ‘light’. Error bars show 95% confidence intervals.

Next, to examine how semantic cues affect sensorimotor prediction, we analysed the fingertip forces applied during the initial lifts of the heavy pair of objects (i.e., the first two experimental trials). As was observed with the perceptual results, the objects’ labels did not create any significant differences in grip (*t*(34) = 1.83, *p* = .076, *d* = .309; Figure 3A) and load (*t*(34) = 0.95, *p* = .352, *d* = 0.160; Figure 3B) force rates. These findings thus provide no evidence that the labels ‘light’ and ‘heavy’ influence initial sensorimotor prediction. The fingertip force rates across all trials are visualised in Figures 4A, 4B, 4C and 4D.

**Figure 5:**
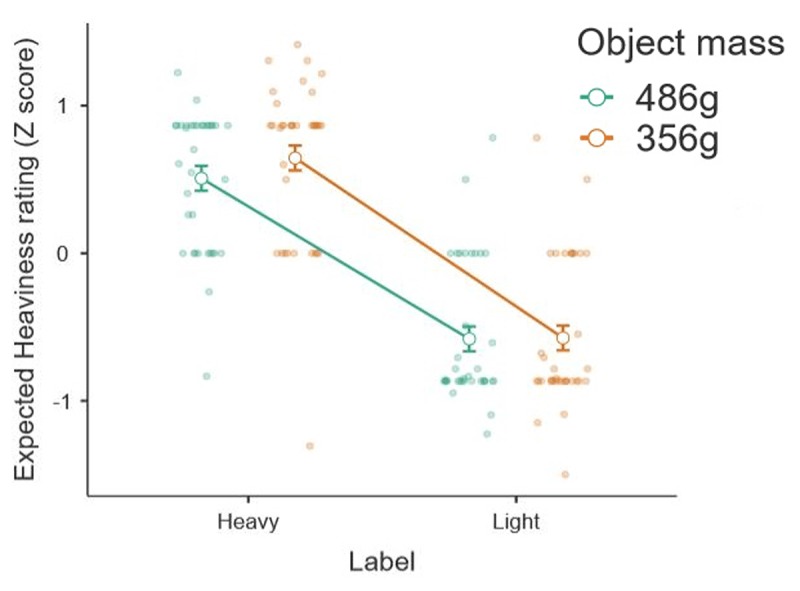
The average pre-lift-off perceptual ratings of expected heaviness, normalized to a z distribution, for each of the four lifted objects.

As expected, when analysing the pre-lift-off judgements of heaviness we found no main effect of mass (*F*(1, 35) = 1.10, *p* = .301, \eta _p^2 = .031) or interaction effects of mass and label (*F*(1, 35) = 1.35, *p* = .253, \eta _p^2 = .037), because there were no visual cues of the differences in mass. By contrast, the labels on the objects did produce explicit expectations prior to physical interaction, such that we observed a significant effect of object label in these pre-lift-off ratings (*F*(1, 35) = 65.06, *p* <.001, \eta _p^2 = .650), with participants expecting the objects labelled as ‘heavy’ to weigh more than the objects labelled as ‘light’ (‘Heavy’: *M* = .58, *SD* = .52; ‘Light’: *M* = –.58, *SD* = .49; Figure 5).

Finally, we examined whether the pre lift-off expected heaviness ratings were related to the post lift-off experienced heaviness ratings, for each individual object. We observed that the pre lift-off expected heaviness ratings did not correlate with the post-lift perceptions of heaviness for the 356 g object labelled ‘light’ (*r* = .049, *p* = .776); ‘heavy’ 356 g object (*r* = .055, *p* = .750); ‘heavy’ 486 g object (*r* = .044, *p* = .799). We did observe a marginal correlation between these measures for the ‘light’ 486 g object (*r* = –.338, *p* = .044). In addition, the pre-lift-off perceptions of heaviness did not influence sensorimotor prediction either. No significant correlation was found between the reported expected heaviness of the 486 g objects and the grip force applied on the initial lift of each 486 g object (‘heavy’ 486 g object: *r* = .249, *p* = .148; ‘light’ 486 g object: *r* = –.161, *p* = .356), or the load force used when lifting the objects (‘heavy’ 486 g object: *r* = .232, *p* = .181; ‘light’ 486 g object: *r* = –.254, *p* = .140).

Overall, this final analysis does not provide any compelling evidence that explicit expectations created by the labels drive subsequent perceptual or sensorimotor judgements.

### Exploratory analyses

**Figure 6:**
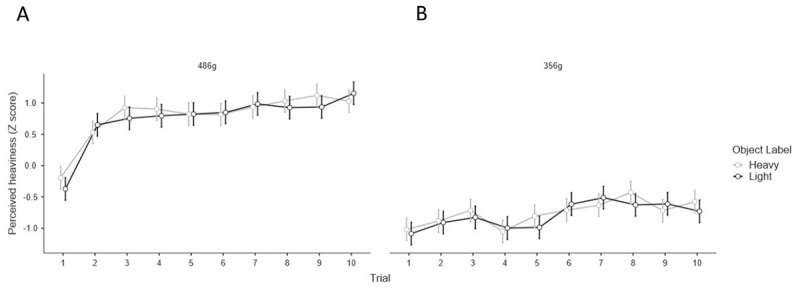
**A.** Perceived heaviness, normalised to a z distribution, across all trials for the 486 g objects labelled ‘heavy’ and ‘light’ and **B.** the 356 g objects labelled ‘heavy’ and ‘light’. Error bars show 95% confidence intervals.

Due to participants frequently commenting that they ignored the labels after the initial lifts, in addition to our planned analysis we also examined whether there was a label weight illusion on the first two experimental trials, with a paired-sample t-test mirroring our analysis of the sensorimotor prediction above. We found no significant differences between the reported perceived heaviness on the first lift of the 486 g object labelled ‘heavy’ and the 486 g object labelled ‘light’ (*t*(35) = 1.40, *p* =.172, *d* = 0.233. The effect of the labels on perception is visualized across each lift, for each set of object weights, in Figure 6A and 6B. Therefore, it seems that the explicit expectations created by the objects did not induce a weight illusion even upon the first interactions, in which participants claimed to be using the semantic cues’ information.

## Discussion

The current study aimed to examine whether semantic cues may impact perceptions of heaviness and sensorimotor prediction when lifting objects and judging their weights. It is well established that the size of an object creates expectations about the object’s weight, and that these expectations subsequently affect how heavy it feels – a phenomenon known as the size-weight illusion (Buckingham, 2014). The SWI is an example of how low-level stimulus properties, such as size, influence the interpretation of incoming sensory information. This study focussed on the impact of higher-level expectations, to investigate whether a novel weight illusion similar to the SWI could be induced by semantic cues. To test this hypothesis, participants reported the felt heaviness of equally-weighted sets of objects labelled as either ‘heavy’ or ‘light’ over multiple lifts. Grip and load force rates were also recorded to understand how the labels influenced sensorimotor prediction upon the initial interactions with the objects. Contrary to the hypothesis, neither perceptions of heaviness nor grip and load force rates were significantly affected by the labels on the objects, despite the fact that these labels influenced explicit pre-lift-off expectations. We thus found no evidence for the proposition that higher-level expectations, derived from semantic cues to mass, affect perception or drive sensorimotor prediction.

These findings highlight the segregated nature of different types of expectation – a term with increasingly variable meaning. This segregation is not simply a low-level vs high-level distinction, as a range of other types of ostensibly high-level expectations have been shown to influence perception of heaviness. The most reliable example comes from the material-weight illusion, whereby objects which appear to be made from low-density materials feel heavier than equally-weighted objects which appear to be made from high-density materials. This illusion, when tactile cues are matched across stimuli, can be induced only by learned associations between the appearance of a material and its usual mass (Buckingham et al., 2009, 2011a). Despite this, the MWI is typically far smaller than the SWI, and the SWI is more consistent across inducing modalities (Saccone et al., 2019). Therefore, it appears that higher-level expectations have a less robust influence on heaviness perception than low-level stimuli, perhaps because they require more inference from the perceiver, and thus have a higher potential for error. Consequently, it is not surprising that different types of higher-level expectations have variable influences on perceptual experiences and motor output.

Although the labels attached to the objects did not influence experienced weight, a significant effect was found on the expected heaviness prior to lifting. In other words, the semantic cues seemed to create explicit expectations about the weight of the objects, however, these explicit expectations did not then influence subsequent perceptions of heaviness upon interacting with the objects, consistent with recent work by Vicovaro and colleagues (2019). These findings align with other research that found that implicit, rather than explicit expectations drove weight illusions. Buckingham and MacDonald (2016) found that prior expectations reflected information about the object’s identity, whereas the subsequent perception of heaviness was influenced by object size; this suggests that implicit expectations derived from size information have a dominating influence on perception, compared to explicit expectations and higher-level inferences. The notion that size has a dominating influence over higher-level expectations when informing perceptual experience of weight is further supported by Vicovaro and Burigana (2017): material, a higher-level expectation, contributed more than size to expected weight, however size contributed more to actual perceived weight (see also Saccone et al., 2019). Similar patterns were found when analysing sensorimotor prediction, suggesting that fingertip forces during object interactions are also primarily driven by implicit expectations (Buckingham and Goodale, 2013).

Accordingly, we propose that in the present study, the (identical) size of each object created implicit expectations about each object’s weight being the same, and this implicit expectation dominated any explicit expectations created by the labels. Presumably the explicit expectations induced by the labels were considered, at a perceptual level, less reliable than analogous material cues which have been shown to induce weight illusions with equally-sized stimuli. Not only is this explanation consistent with a recent review article proposing that size cues are fundamentally distinct from other types of cues (Saccone and Chouinard, 2019), it is further substantiated by participants’ informal feedback. Many participants commented that they suspected the objects would be similar weights because they were the same size, and that they consciously ignored the labels on the objects after the initial lifts when they realised that size provided more reliable information. We did, however, see no evidence of a greater magnitude of illusion on early trials (Figure 6). Consequently, the overall pattern of results in the present study could be explained by the implicit low-level expectations dominating any influence of the higher-level explicit expectations on perception of heaviness and sensorimotor prediction.

The pattern of initial fingertip force application in this study also highlights the disassociation between conscious expectations and sensorimotor prediction when first encountering the object. This finding has important applied consequences for our real-world interaction with objects. Labels such as ‘light’ and ‘heavy’ are often used to act as signals to inform individuals’ interactions with objects (e.g., labelling suitcases this way for baggage handlers at airports). Here, however, we found no evidence that labels influenced the way that objects were gripped and lifted, questioning the effectiveness of such methods. Of course, it is possible that any differences may be too small for the current work to detect (which was powered only to examine a perceptual effect), and/or may only manifest in different types of grip. Future work should examine whether this effect holds in contexts where weight differences are explicitly expected, or have more dramatic consequences (e.g., airports or post offices).

The present findings help to understand how the segregated nature of higher-level expectations creates variability in how prior knowledge influences perceptions of heaviness and sensorimotor prediction. Whilst this study found that semantic cues did not create illusory weight differences, other studies have found that higher-level expectations such as materials and object identity can in fact produce weight illusions, albeit less-robustly than size cues. Accordingly, perhaps the use of the term ‘expectations’ is too broad, as it appears that many types of priors can be formed, and that they may influence our perception in different ways. Therefore, it may be more useful to further investigate how more specific categories of high-level expectations influence perception and action, to help clarify the term ‘expectations’. As it is clear that the mechanisms underlying how higher-level expectations interact with sensory input are separate from those of low-level stimulus, more research is needed to understand how different types of information can influence perception and action in the world.

## Data Accessibility Statement

Raw data, and the preregistered analysis plan, can be found at: https://osf.io/ug3hc/.
